# Resveratrol Improves Cognitive Function in Sleep‐Deprived Mice by Inhibiting p38 MAPK Signaling Pathway

**DOI:** 10.1002/fsn3.70816

**Published:** 2025-09-26

**Authors:** Yijie Tang, Xiyuan Xie, Haixing Wu, Xinlei Huang, Jiapeng Qiu, Yupeng Han, Peng Ke, Kai Zeng, Xiaodan Wu

**Affiliations:** ^1^ Department of Anesthesiology, Fuzhou University Affiliated Provincial Hospital, School of Medicine Fuzhou University Fuzhou Fujian China; ^2^ Department of Anesthesiology The First Affiliated Hospital of Fujian Medical University Fuzhou China

**Keywords:** cognitive function protection, MAPK signaling pathway, p38, resveratrol, sleep deprivation

## Abstract

Resveratrol (RES) is a naturally occurring polyphenolic compound possessing neuroprotective properties. This study aims to examine the potential of RES to ameliorate sleep disorder‐associated cognitive deficits induced by sleep deprivation (SD) in mice. Adult male C57/BL6 mice were subjected to SD for 72 h, and RES were administrated. Behavioral tests were performed to quantify the cognitive behaviors. RNA‐sequencing coupled with bioinformatic analysis was executed to examine gene expression patterns in the hippocampus. Furthermore, the levels of apoptosis and synaptic plasticity were measured by western blotting and histological staining techniques, including the TUNEL and Nissl methods. Morris water maze, novel object recognition and open field tests demonstrated that RES swiftly attenuated the cognitive impairment induced by SD. RNA‐sequencing indicated that the mitogen‐activated protein kinase (MAPK) pathways were significantly downregulated by RES administration following SD. Additionally, the impaired synaptic plasticity and apoptosis elicited by SD in the hippocampus were improved by RES. SD causes cognitive dysfunction and hippocampal neuronal damage in mice. Treatment with RES improves cognitive function and attenuates neuronal damage in the hippocampus of sleep‐deprived mice. Furthermore, the MAPK signaling pathway has been identified as a pivotal target in these neuroprotective effects. These findings will contribute to providing theoretical support for dietary supplementation of RES to prevent or improve cognitive dysfunction caused by sleep problems.

## Graphical abstract

Sleep disorder leads to cognitive impairment. Therefore, finding a natural substance to improve cognitive problems caused by sleep disorders is particularly important. Resveratrol (RES), a naturally occurring polyphenolic compound possessing neuroprotective properties, has demonstrated the ability to enhance cognitive function. Finally, we found that SD causes cognitive dysfunction and hippocampal neuronal damage in mice. Treatment with RES improves cognitive function and attenuates neuronal damage in the hippocampus of sleep‐deprived mice. Furthermore, the MAPK signaling pathway has been identified as a pivotal target in these neuroprotective effects.

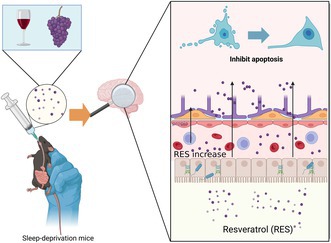

**Abbreviations**
DMSO: dimethyl sulfoxide
MAPK: mitogen‐activated protein kinase
OFT: open field test
RES: resveratrol
SD: sleep deprivation

## 1 Introduction

Sleep is a ubiquitous physiological phenomenon in humans and animals. It is integral to various brain functions, including neurobehavior, cognition, memory consolidation, emotion regulation, pain regulation, and clearance of brain metabolites (Watson et al. 2015). However, the rapid pace of societal development and escalating life pressures have led to a significant increase in the prevalence of sleep deprivation (SD) worldwide. SD, a serious sleep disorder resulting from disruptions in the circadian rhythm and imbalances in sleep homeostasis, can have detrimental effects on human health. These effects encompass a range of conditions, including obesity, stroke, diabetes, cardiovascular disease, and cognitive impairment (Watson et al. 2015; Arora and Taheri 2017; Jiang et al. 2017; Nir et al. 2017; Covassin et al. 2018).

In a population‐based clinical study involving over 10,000 participants globally, approximately 50% of individuals who reported sleeping less than seven hours per night exhibited cognitive decline (Wild et al. 2018). SD is known to have adverse effects on cognitive functions. Neuroimaging studies have revealed scattered changes in frontoparietal control areas, secondary sensory processing areas, and thalamic areas in patients with SD, as compared with controls, suggesting alterations in cognitive function (Lowe et al. 2017). More specifically, an animal study reveals that SD impairs spatial reference memory, spatial working memory, and cue learning in mice (Suzuki et al. 2020). Sleep disorders leading to cognitive decline are a serious public health issue that severely impacts people's quality of life. Currently, the solution to this problem involves using sedative medications to improve sleep. However, long‐term use of sedative drugs can lead to drug tolerance, making it urgent to develop new strategies to prevent and address this issue. Regular dietary supplementation may serve as a potential breakthrough. Therefore, finding a natural substance to improve cognitive problems caused by sleep disorders is particularly important.

Resveratrol (RES), a natural non‐flavonoid polyphenol and phytoalexin especially in grape skin and red wine (Anastácio et al. 2014), possesses a range of bioactive properties, including anti‐inflammatory, anti‐oxidation, and anti‐tumor effects (Pizarro et al. 2011; Bai et al. 2014; Song et al. 2014; Chelsky et al. 2015; Sadi and Konat 2016). RES can increase the expression of presynaptic and postsynaptic proteins in the hippocampus, thereby protecting synaptic plasticity from damage caused by lead exposure in rats (Wang et al. 2021). In a cerebral ischemia model, RES was observed to ameliorate the functional and structural plasticity of synapses in rats with cerebral ischemia (Li et al. 2016), and to upregulate the expression of Bcl‐2 in the hippocampus, thereby counteracting cell apoptosis. These findings suggest its potential as an innovative and effective therapeutic intervention for vascular dementia (Li et al. 2012). Similar beneficial effects have been noted in diabetic rats. Notably, pretreatment with RES can alleviate cognitive dysfunction induced by isoflurane in aged mice, exerting both anti‐inflammatory and anti‐apoptotic effects (Tian et al. 2016).

RES has demonstrated neuroprotective properties across various disease models. Nonetheless, its capacity to ameliorate cognitive impairment induced by SD remains to be elucidated. The present study is designed to investigate the impact of RES on cognitive function in a SD mice model and to delineate the underlying mechanisms. This investigation aims to provide theoretical support for dietary supplementation of RES to prevent or improve cognitive dysfunction caused by sleep problems.

## 2 Materials and Methods

### 2.1 Animals

A total of 180 male C57/BL6 mice, aged 6–8 weeks and weighing approximately 20 g, were purchased from the Laboratory Animal Center of Fujian Medical University. These mice were fed in a clean laboratory of the Laboratory Animal Center of Fujian Medical University at 24°C ± 2°C and humidity of 50%–70%. We simulated the standard day and night environment of 12 h of light (ZT0–ZT12) and 12 h of darkness (ZT13–ZT24), allowing for *ad libitum* access to food and water. The mice acclimated to the environment for 1 week before the experiment. The experimental procedures were all approved by the Experimental Animal Ethics Review and Use Committee of Fujian Medical University and were conducted in accordance with the animal management regulations and rules of Fujian Medical University to maximize animal welfare.

### 2.2 SD Model

SD model was constructed using a sleep deprivation box (Model KW‐BD, Nanjing Darwin Technology Co. Ltd.). The use of the restricted access pole sleep deprivation method avoids the additional physiological burden caused by excessive exercise in animals, while still achieving effective sleep deprivation in the experimental design. Compared to the water platform method, it reduces the animals' stress response because no additional physiological stress is imposed, allowing researchers to more clearly observe the effects of sleep deprivation (Villafuerte et al. 2015; Pires et al. 2021; Krishnan et al. 2025). Inside the sleep deprivation box, the mice were unable to enter the sleep state due to the interference caused by the rotating metal bars. The parameters of the cyclic rotation of the metal bar were set as follows: 12 revolutions/min, and the direction of rotation was changed every 1.5 revolutions. The SD and SD + RES groups began 72 h of continuous acute sleep deprivation after the end of the last day of gavage.

### 2.3 Grouping of Experimental Animals

1. This part aimed to verify the effective concentration of RES in improving cognitive function by dividing mice into the following groups: Control group, SD group, SD +25 mg/kg RES group, SD +50 mg/kg RES group, SD +100 mg/kg RES group, and SD +200 mg/kg RES group. RES (Sigma‐Aldrich, #R5010, ≥ 99% purity) was dissolved in dimethyl sulfoxide (DMSO) and then diluted with saline to a DMSO concentration of 10%. The 14‐day gavage ensures that RES reaches a steady‐state concentration in the body, minimizing the impact of metabolic fluctuations on its efficacy. At the beginning of the experiment on the 1st to 14th day, gastric gavage was performed every day from 9:00 to 10:00 a.m. RES was given in a volume of 0.4 mL, while the Control and SD groups were given an equal volume of solvent by gastric gavage.
2. This part of the experiment aimed to explore the potential mechanisms by which Res improves cognitive impairment following sleep deprivation. The mice were randomized into the Control group, SD group, SD + RES group, and RES group. In the SD + RES group and RES group, RES 100 mg/kg was given in a volume of 0.4 mL, while the Control and SD groups were given an equal volume of solvent by gastric gavage.
3. Anisomycin, as a commonly used stress stimulator, can activate the p38 MAPK signaling pathway. To verify the role of the p38 MAPK signaling pathway in the improvement of cognitive function after sleep deprivation by Res, the remaining mice were randomized into the Control group, SD group, SD + RES group, and SD + RES + Anisomycin group. Mice in the SD + RES + Anisomycin group were fed with anisomycin (2.5 mg/kg/day) from the 15th to 17th days in the making mold period (Li et al. 2010).

### 2.4 Behavioral Tests

#### 2.4.1 Morris Water Maze (MWM)

The Morris water maze is widely used for cognitive function testing based on the rodent's natural aversion to water, and trains experimental animals to use visual cues to locate and search for platforms hidden underwater in order to get out of the water environment. The Morris water maze consists of a 120 cm diameter, 50 cm high black circular pool filled with 30 cm of warm water (constant at 21°C–22°C). The pool was divided into four quadrants and pasted with circular, triangular, square, and star‐shaped stickers as visual cues for mice to find the platform. We set up a disc‐shaped platform about 28 cm high (slightly below the water level) in the middle of the third quadrant. Keep a constant light source in the laboratory. The movements of the mice in the pool were recorded by a camera placed on the ceiling and analyzed using the ANYmaze animal behavior video analysis system.

The specific steps contain two parts—place navigation test and spatial probe test. a. Place navigation test: (a) test was conducted for 4 days, with mice placed in a pool with their heads facing the wall of the tank from each quadrant each day. The camera stopped recording after the mice climbed onto the platform or after more than 60 s. If the mice did not find the platform within 60 s, they were guided to the platform and allowed to stay on the platform for 15 s before conducting the place navigation test in the next quadrant. The place navigation test in four quadrants was carried out in turn every day. After the end of the experiment on the fourth day, mice in the SD group and SD + RES group were transferred to the sleep deprivation box for 3 days of acute sleep deprivation. (b) Spatial probe test: all four groups completed the spatial probe test within 2 h after sleep deprivation. The platform was removed, and the mice were placed into the water from the entry point of the first quadrant facing the wall of the pool, and the number of times the mice crossed the range of the original platform and the residence time in the target quadrant were recorded within 60 s. The mice were monitored by a video camera above the water maze. The whole process was monitored by a video camera above the water maze, and the data were analyzed and processed using the Behavioral Trajectory Tracking System software.

#### 2.4.2 Open Field Test (OFT)

OFT is a method to evaluate the autonomous behavior, exploration behavior, and tension of experimental animals in a new environment. The experimental apparatus consists of an open field reaction chamber and the ANYmaze video analysis system. The mouse open field reaction chamber is 50 cm long, 50 cm wide, and 40 cm high. The bottom of the chamber is white. The bottom surface of the box is divided into 25 cells, from the center point outward, setting the central area and the surrounding area. All four groups completed OFT within 2 h after sleep deprivation. Taking the animal out, facing away from the experimenter, it is placed in the center point and left immediately. The experiment is timed for 5 min. The video analysis system recorded the mouse activity track, rest time, central region movement time, and central cell crossing times. After each mouse was tested, the stool in the box was cleaned, and anhydrous ethanol was sprayed to remove the odor of the previous mouse.

#### 2.4.3 Novel Object Recognition (NOR)

NOR test was employed to evaluate the spatial learning and memory capabilities of mice, focusing on their ability to recognize novel objects. Habituation Phase: 2 days prior to testing, mice were placed in the behavioral laboratory to acclimate to the environment. Training Phase: 1 day before testing, two identical objects (in shape, size, and color) were positioned near one wall within an open‐field arena. Mice that had completed the habituation phase were reintroduced into the arena, placed equidistant from the two objects. They were allowed to freely explore the arena for 10 min. During this period, their movement trajectories and sniffing behaviors were recorded. After each session, the arena and objects were thoroughly cleaned with 70% ethanol to eliminate residual odors. Testing Phase: On the day of testing, one of the familiar objects was replaced with a novel object differing in size and color, while the other object remained unchanged. The positions of both objects were switched to the opposite sides of the arena from their original locations. Mice were placed in the center of the arena, equidistant from both objects, with their heads facing away from them. Exploration time and frequency directed toward the familiar and novel objects—using the mouth or forelimbs—were recorded. After each test, the arena and objects were cleaned with 70% ethanol to remove residual odors.

### 2.5 Specimen Collection

Mice were anesthetized with 0.02 mL/g intraperitoneal injection of 0.3% pentobarbital sodium. When the eyelash and corneal response were weakened, muscle tension was reduced, and the forceps toe reflex was weakened, the mouse was placed in the sample tray in the supine position.

### 2.6 Nissl Staining

Rapidly open the chest cavity and pericardium of mice, expose the heart, cut a small hole in the right auricle, and immediately perfuse with saline (flow rate of about 10 mL/min), first fast and then slow, until the color of the blood became clear, the lungs whitened, and the liver gradually changed from dark red to gray‐yellow, at which time, 20 mL of 4% paraformaldehyde solution was perfused into the heart for about 2 min; after the end of the perfusion, open the skull of the mouse, take out the brain tissue intact, and immerse it in 4% paraformaldehyde fixative, and fix it at 4°C for 24 h. The mouse was then fixed for 24 h at 4°C. We removed the brain tissue and after ethanol dehydration, the ethanol was removed with xylene and made transparent. The soft wax was soaked and embedded in paraffin wax, cooled at −20°C, and then the cooled wax blocks were serially sliced in the coronal position with a paraffin slicer, with a thickness of about 4 μm. The slices were attached to slides and baked in an oven at 60°C for spare use. The slices were dewaxed by xylene and anhydrous ethanol, rinsed with distilled water, and then stained with 1% toluidine blue for 40 min (or tar violet staining for 30 s) in a 60°C oven. After washing the dye with distilled water, the slices were rehydrated, retransparent, and finally sealed with neutral gum. The slices were examined microscopically and observed under low and high magnification, and the pathological changes in the CA3 region of the mouse hippocampus were photographed at 20 × 10× and the percentage of neuronal damage in the CA3 region was counted.

### 2.7 Transcriptome Sequencing

The mice were anesthetized and then their cervical vertebrae were dislocated and executed, and their brains were severed, and the mouse hippocampus was rapidly isolated on ice, and the obtained hippocampal tissues were placed in pre‐labeled cryopreservation tubes and stored in a −80°C refrigerator. Sequencing was entrusted to Upper Guangzhou Kidio Biotechnology Service Co. After RNA extraction using the TRIzol method and other techniques, its purity (OD260/280 between 1.8 and 2.2 and OD260/230 > 2.0), integrity (RIN ≥ 7 for mammals), and concentration (≥ 50 ng/μL) were assessed. rRNA was removed by conventional kits, and mRNA was enriched. The enriched mRNA was further reverse transcribed to form double‐stranded cDNA, reverse transcription was performed at 55°C for 60 min. After repairing the double ends of cDNA (20°C) and adding the junction (overnight at 22°C), PCR amplification was performed to construct the on‐line library. In order to ensure the data quality, data filtering was performed on the raw data before information analysis to reduce the analysis interference brought by invalid data. Firstly, the raw reads were quality‐controlled by fastQC to filter the low‐quality data and obtain clean reads. The base quality is required to be Q30 > 85%, and the Trimmomatic trimming parameters are set as: SLIDINGWINDOW: 4:15 and MINLEN: 50. The clean reads were compared to the ribosomal database of the species using the short‐reads matching tool bowtie2, and the unmapped reads were retained to be used for the subsequent transcriptome analysis, and the mismatched reads were removed. Reads were retained for subsequent transcriptome analysis. The high‐quality data were used to carry out mouse genome‐based alignment analysis using HISAT2 software. Based on the comparison results of HISAT2, we reconstructed the transcripts using Stringtie and calculated the expression amount of all genes in each sample using RSEM. The expression amounts were presented as raw reads count and FPKM, and gene expression differential analysis was performed using DESeq2 software. |log2FC| ≥ 1.5 with *p* < 0.05 was used as a criterion to define differential genes. Functional categorization of the resulting differential genes helps to further understand their biological roles. The differential genes were uploaded to Metascape database for GO functional annotation and KEGG pathway analysis, and the top entries with *p* < 0.05 were selected for the presentation of results and experimental verification.

### 2.8 Western Blot

The brain tissue was lysed using RIPA buffer containing protease and phosphatase inhibitors on ice for 30 min, with vortexing every 10 min. The lysates were then centrifuged at 12,000 rpm for 10–15 min, and the supernatants were collected. Protein concentrations were determined using a BCA protein assay kit. For each sample, 20–40 μg of total protein was mixed with loading buffer containing SDS and β‐mercaptoethanol, denatured at 100°C for 15 min, and then loaded onto a polyacrylamide gel. Electrophoresis was performed at a constant voltage (80 V for stacking gel, then 120 V for resolving gel). Proteins were transferred to PVDF membranes using a wet transfer system at 220 mA for 1.5 h (PVDF membranes were pre‐activated in methanol prior to transfer). Membranes were blocked in TBST containing 5% non‐fat milk or BSA at room temperature for 1 h with gentle shaking to reduce non‐specific binding. Membranes were then incubated overnight at 4°C with primary antibodies diluted at 1:1000. The next day, membranes were washed three times with TBST for 5–10 min each, followed by incubation with HRP‐conjugated secondary antibodies (diluted 1:10,000) at room temperature for 1 h. After washing again three times with TBST, the membranes were developed using enhanced chemiluminescence (ECL) reagents, and protein bands were visualized and analyzed using an imaging system.

### 2.9 Tunnel Staining

The brain was gathered and then fixed in 10% formalin neutral solution, trimmed, and embedded in paraffin. Sections (5 mm thick) for terminal deoxynucleotide transferase (TdT) mediated nick end labeling (TUNEL) were fixed in a 4% solution of paraformaldehyde. TUNEL analysis was performed with an ApopTag peroxidase in situ apoptosis detection kit (Chemicon International Inc., USA) according to the manufacturer's instructions.

### 2.10 Statistical Analysis

SPSS 26.0 software was used for statistical analysis of data. Continuous variables conformed to normal distribution and were expressed as mean ± standard deviation (*x* ± *s*) with the chi‐square Levene test. Continuous variables not conforming to normal distribution were expressed as median (quartiles) (M (P25, P75)). Comparisons between groups of data that conformed to normal distribution and were consistent with chi‐square were analyzed by one‐way ANOVA; post hoc comparisons were tested by the LSD method, and comparisons between groups that did not conform to chi‐square were tested by Dunnett's T3 corrected test. The chi‐square test/Fisher's exact probability method was used for count data. The test level *α* = 0.050 was specified, and *p* < 0.05 was considered statistically significant.

## 3 Results

### 3.1 One Hundred Milligrams Per Kilogram RES Pretreatment Improves Cognitive Function in Sleep‐Deprived Mice

**FIGURE 1:**
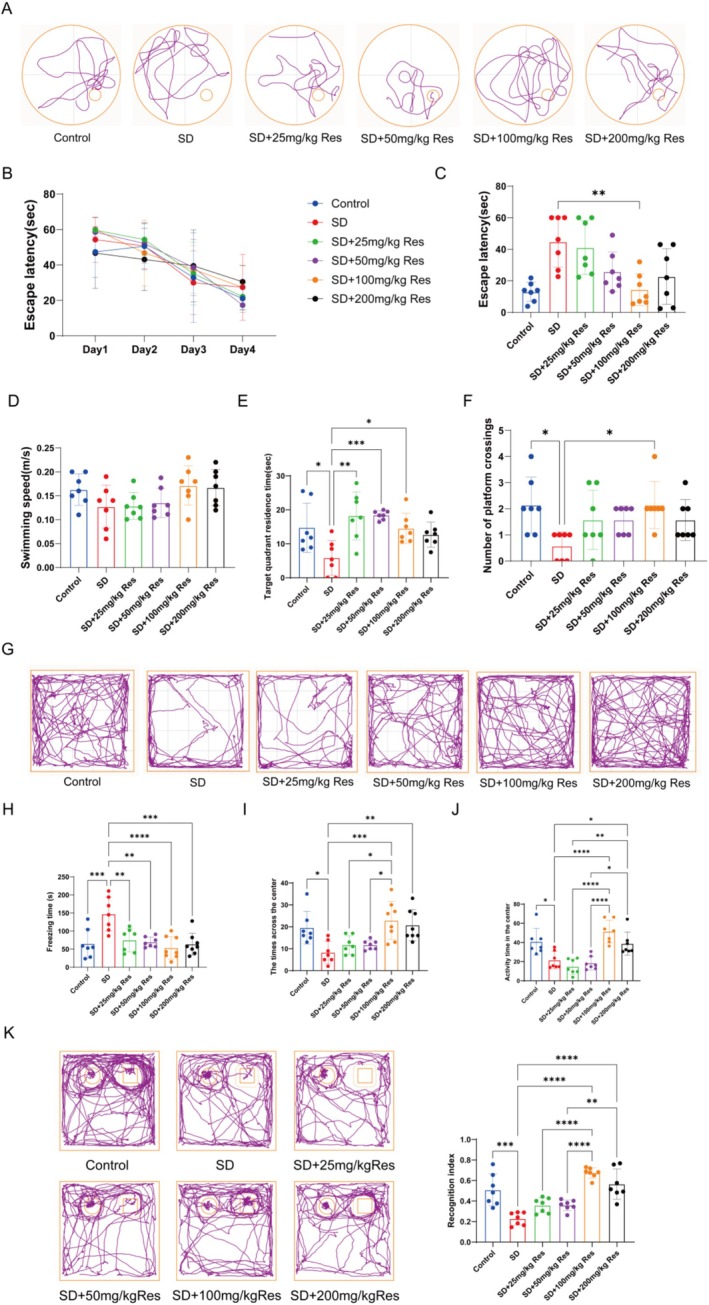
SD induced cognitive impairment and behavioral test results after RES treatment. (A) Typical trajectory diagram of MWM test. (B–F) The results of MWM test. (G) Typical trajectory diagram of OFT. (H–J) The results of OFT. (K) The results of NOR. The left “○” represents the old object, and the right “□” represents the novel object (*n* = 7; *p < 0.05, **p < 0.01, ***p < 0.001, and ****p < 0.0001).

The Morris water maze test was employed to assess the learning and memory capabilities of mice. In comparison to the Control group, mice in the SD group exhibited erratic movement trajectories, predominantly skirting the maze's perimeter, and showed diminished passage through the target quadrant along with fewer instances of crossing the original platform (Figure 1A). Conversely, mice in the SD +100 mg/kg RES group displayed movement patterns akin to the Control group, characterized by purposeful navigation primarily within the target quadrant and increased platform crossings. Notably, locomotor abilities did not differ significantly among the groups. In the SD group, the time spent in the target quadrant was reduced, accompanied by a decline in platform crossings. However, pretreatment with 100 mg/kg RES extended the residence time in the target quadrant and augmented platform crossings (Figure 1B–F).

As shown in Figure 1G–J, analysis of trajectory from the open field test revealed that, in contrast to the Control group, the SD group exhibited a propensity for peripheral movement, with minimal traversal of the central area. Conversely, both SD +100 mg/kg RES and SD +200 mg/kg RES groups mirrored the Control group's behavior, displaying distributed movement trajectories across all areas and increased entry into the central zone. The novel object recognition test was used to assess the spatial learning and memory abilities of mice, particularly their ability to recognize novel objects. Analysis of the novel object recognition index showed that mice in the SD group did not exhibit a significant preference for the novel object and spent less time exploring it. Mice treated with 100 and 200 mg/kg RES showed a stronger interest in exploring the novel object (Figure 1K).

Sleep deprivation resulted in impaired learning and memory in mice, alongside alterations in anxiety and depression levels. However, 100 mg/kg RES already demonstrated the potential to enhance cognitive function.

### 3.2 Explored the Possible Mechanism of RES Pretreatment Improving Cognitive Function in Sleep‐Deprived Mice by Transcriptome Sequencing

**FIGURE 2:**
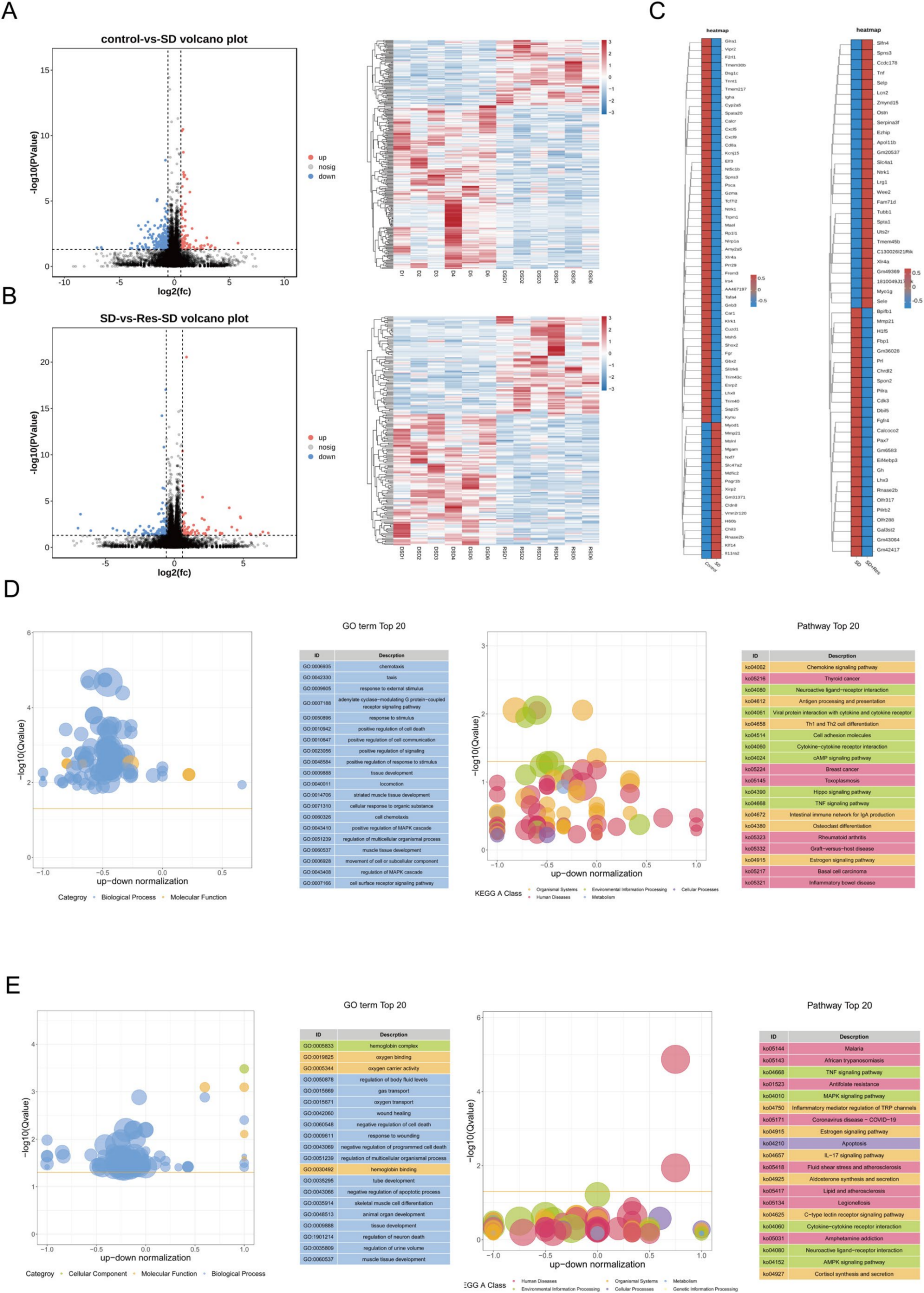
Differentially expressed gene analysis in SD versus control and SD + RES versus SD. (A, B) Heatmap and volcano plot showing differentially expressed genes with *p* < 0.05 and |log2FC| > 1.5 between SD versus control and SD + RES versus SD mice. (C) Heatmap showing the most differentially expressed genes filtered by FDR ≤ 0.01 and effect size> ±0.5 in each cohort. (D) Bubble graphs showed the enriched GO terms (Left) and KEGG pathway terms of DEGs in SD mice compared to control mice. (E) The top 15 enriched GO (Left) and KEGG pathway terms (Right) ranked by the number of DEGs in SD + RES mice compared to SD mice. *n* = 6.

RNA‐Seq sequencing analysis was performed on hippocampal tissues of three groups of mice. Using |log2FC| ≥ 1.5 and *p* < 0.05 as the standard to screen differential genes, the results showed that there were 273 genes significantly differentially expressed between the SD group and the Control group, of which 78 genes were up‐regulated and 195 genes were down‐regulated in the SD group (Figure 2A). Compared with the SD group, 185 differential genes were found in the SD + RES group, of which 106 genes were up‐regulated and 79 genes were down‐regulated in the SD + RES group (Figure 2B). Heatmap representations are shown for differentially regulated genes based on FDR (≤ 0.01) and standardized effect size (absolute value > ±0.5) to identify those genes most strongly affected by sleep deprivation and RES treatment (Figure 2C). The overlap analysis revealed 29 significant DEGs, detailed examination of the overlapping genes were transcriptionally reversed by RES treatment (Table S1).

To further understand the biological function of DEGs induced by SD and RES treatment, the GO and KEGG enrichment analysis was performed. It is found that the genes upregulated in the SD group (Figure 2D) and downregulated in the SD + RES group (Figure 2E) were mainly involved in the MAPK signaling pathway and the regulation of apoptosis. These results suggest that the potential mechanism by which RES improves cognitive impairment induced by SD may be related to MAPK signaling pathway, hippocampal neuronal cell death.

### 3.3 RES Reduces Hippocampal Neuronal Damage in Sleep‐Deprived Mice

**FIGURE 3:**
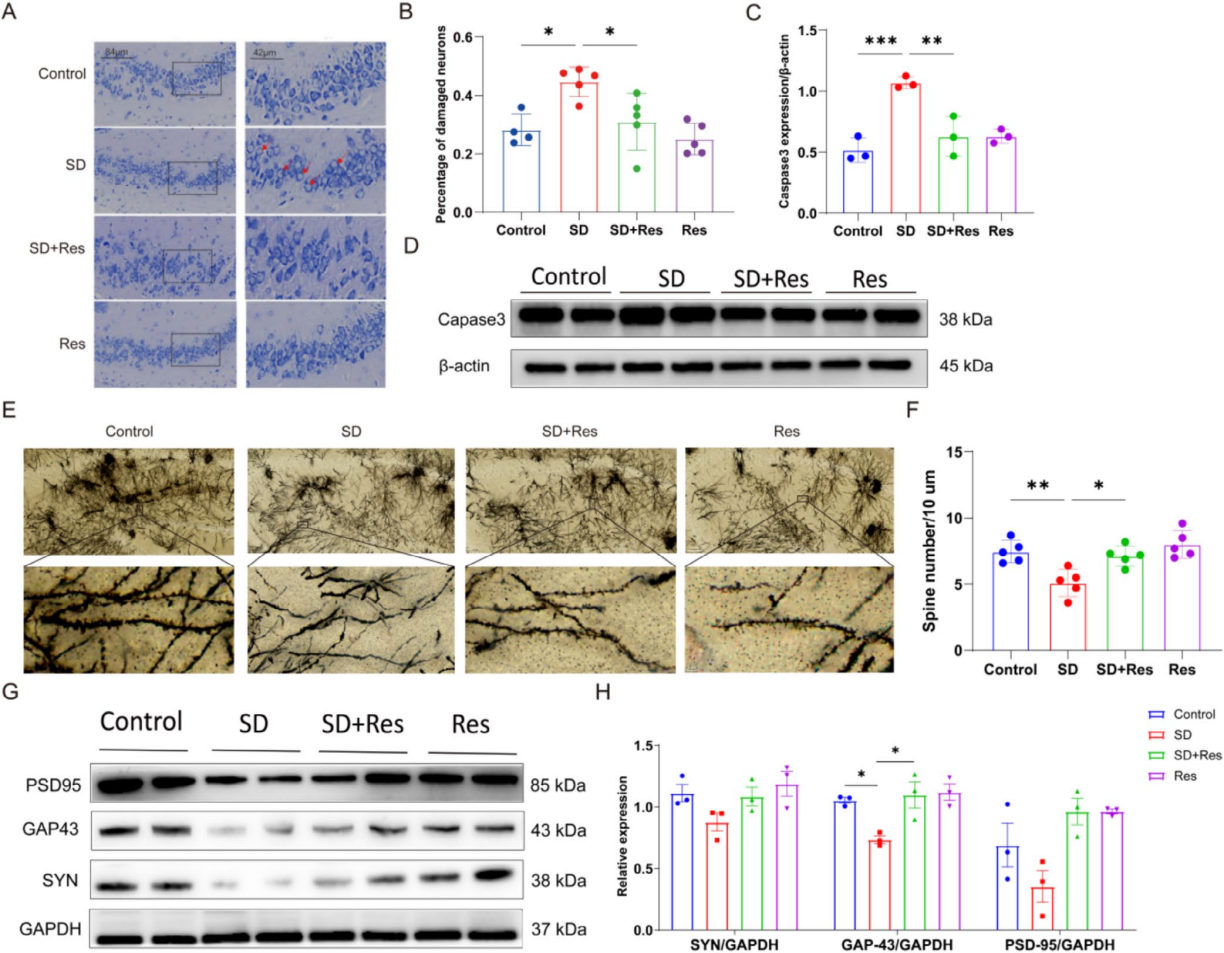
Hippocampal neuronal damage in SD mice were restored by RES treatment. (A, B) The results of Nissl Staining. Compared with the control group, the SD group showed mild changes in neuronal morphology, including swelling of the cytosol, deep staining of the nucleus, lighter Nissler staining (*n* = 4). (C, D) The results of apoptosis‐associated proteins expression. Caspase‐3 expression was upregulated in the SD group compared to the control group, RES reversed this result (*n* = 3). (E, F) RES improves synaptic plasticity in CA3 region of sleep‐deprived mice (*n* = 5). (G, H) The results of synaptic plasticity‐related protein expression. GAP‐43 expression was downregulated in the SD group compared to the control group, RES reversed this result (*n* = 3; *p < 0.05, **p < 0.01, and ***p < 0.001).

We found that SD caused damage in the morphology of hippocampal neurons. The neuronal cell body was swollen, the nucleus was dark and deviated, the Nissl staining became lighter, the cells were more transparent white, and the percentage of damaged cells in the CA3 region was increased. Pretreatment with RES significantly alleviated the above morphological changes, and the percentage of damaged cells in the CA3 region was also significantly reduced (Figure 3A,B). The biomarker of apoptosis Caspase‐3 was upregulated in the SD group compared to the control group, RES reversed this result (Figure 3C,D). SD reduced the density of dendritic spines in hippocampal CA3 pyramidal neurons of mice, and RES can significantly alleviate the damage of neuronal structure (Figure 3E,F). The results showed that sleep deprivation decreased the expression of synapse‐associated proteins GAP‐43 in hippocampus of mice, and RES pretreatment could prevent this effect (Figure 3G,H). These results suggest that sleep deprivation can cause hippocampal neuronal damage, and RES can prevent this.

### 3.4 Pretreatment With RES Inhibits p38 MAPK Activation in Sleep‐Deprived Mice

**FIGURE 4:**
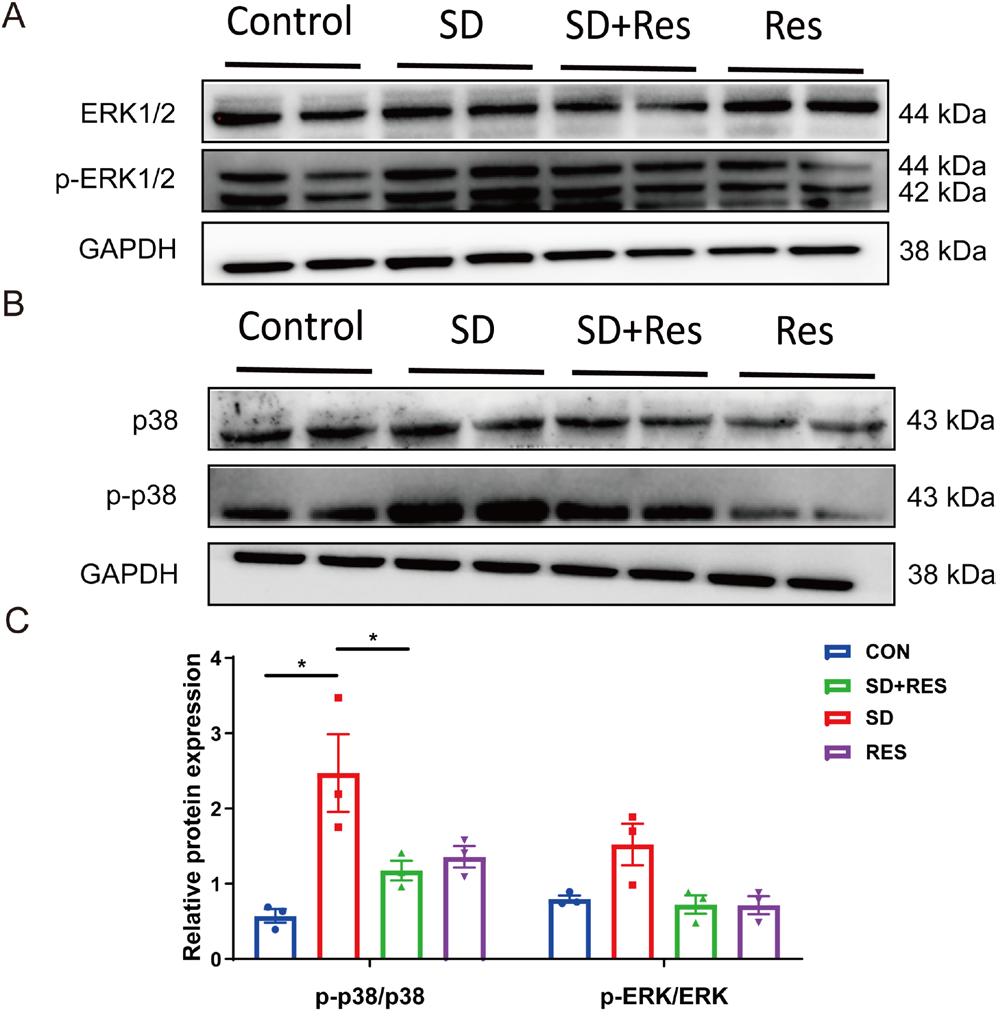
The results of MAPK signaling pathway expression. (A–C) Compared with the control group, the expression of p‐p38 in the SD group increased. Compared with the SD group, the expression of p‐p38 in SD + RES group decreased (*n* = 3; *p < 0.05).

The sequencing results showed that there were differences in the two branches of the MAPK signaling pathway, p38 and ERK. As shown in Figure 4, p‐p38/p38 was increased in the SD group and decreased in the SD + RES group. The results showed that the expression of phosphorylated p38 MAPK was increased in the hippocampus of sleep‐deprived mice, indicating that the MAPK pathway was activated, and RES pretreatment could inhibit the p38 MAPK signaling pathway. The MAPK signaling pathway may be a potential target for RES to improve cognition in sleep‐deprived mice.

### 3.5 Anisomycin Attenuats the Effect of RES on the Inhibition of p38 MAPK Signaling Pathway Activation

**FIGURE 5:**
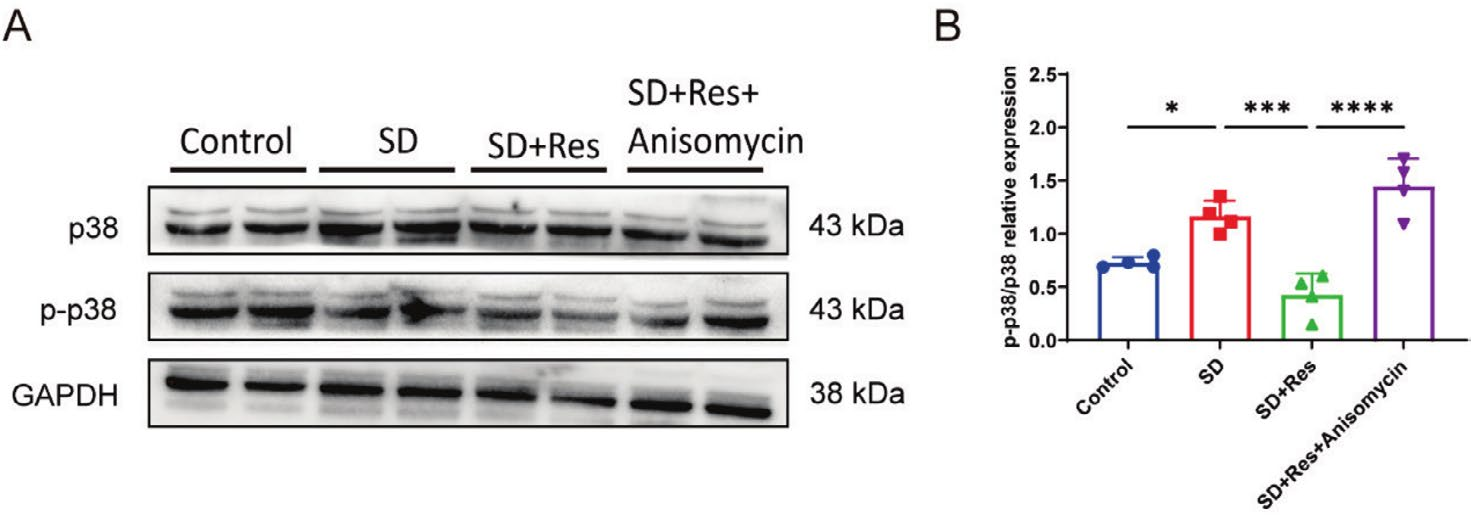
Anisomycin activated p38 MAPK signaling pathway. RES inhibited the activate ion of p38 MAPK signaling pathway in the hippocampus of sleep‐deprived mice, while anisomycin reversed this effect (*n* = 4; *p < 0.05, ***p < 0.001, and ****p < 0.0001).

To further clarify the effect of RES by inhibiting the p38 MAPK signaling pathway, intraperitoneal injection of anisomycin was used to verify this idea. The results showed that RES inhibited the activation of the p38 MAPK signaling pathway in the hippocampus of sleep‐deprived mice, while anisomycin reversed this effect (Figure 5).

### 3.6 RES Improves Cognitive Function in Sleep‐Deprived Mice by Inhibiting p38 MAPK Signaling Pathway

**FIGURE 6:**
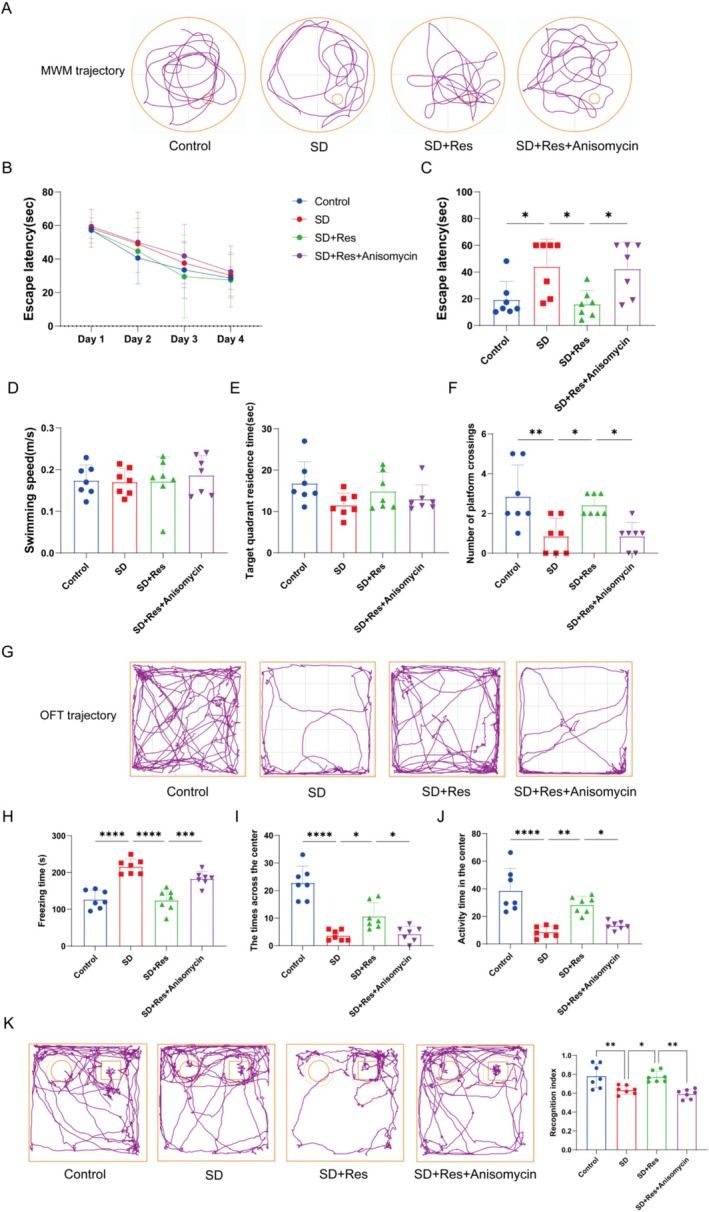
RES improves cognitive function in sleep‐deprived mice by inhibiting the p38 MAPK signaling pathway. (A) Typical trajectory diagram of the MWM test. (B–F) The results of the MWM test. (G) Typical trajectory diagram of the OFT. (H–J) The results of the OFT. (K) Trajectory plots and cognitive index comparison of four groups of mice in the novel object recognition test. The left ‘○’ represents the old object, and the right ‘□’ represents the novel object (*n* = 7; *p < 0.05, **p < 0.01, ***p < 0.001 and ****p < 0.0001).

As shown in Figure 6A–F, mice in the SD group exhibited erratic movement trajectories, predominantly skirting the maze's perimeter, and showed diminished passage through the target quadrant along with fewer instances of crossing the platform. Conversely, mice in the SD + RES group displayed movement patterns akin to the Control group, characterized by purposeful navigation primarily within the target quadrant and increased platform crossings. Notably, locomotor abilities did not differ significantly among the groups. In the SD + RES + Anisomycin group, the time spent in the target quadrant was reduced, accompanied by a decline in platform crossings, and their movement trajectories were similar to those in the SD group.

In contrast to the Control group, the SD group exhibited a propensity for peripheral movement, with minimal traversal of the central area. Conversely, both the SD + RES groups mirrored the Control group's behavior, displaying distributed movement trajectories across all areas and increased entry into the central zone. However, anisomycin reversed the behavioral improvement induced by RES in sleep‐deprived mice (Figure 6G–J).

Analysis of the novel object recognition index showed that mice in the SD group did not exhibit a significant preference for the novel object and spent less time exploring it. Mice treated with RES showed a stronger interest in exploring the novel object. Anisomycin reversed the RES‐induced behavioral improvement in sleep‐deprived mice, indicating that RES acts by inhibiting the p38 MAPK signaling pathway (Figure 6K).

### 3.7 RES Protects Hippocampal Neurons in Sleep‐Deprived Mice by Inhibiting p38 MAPK Signaling Pathway

**FIGURE 7:**
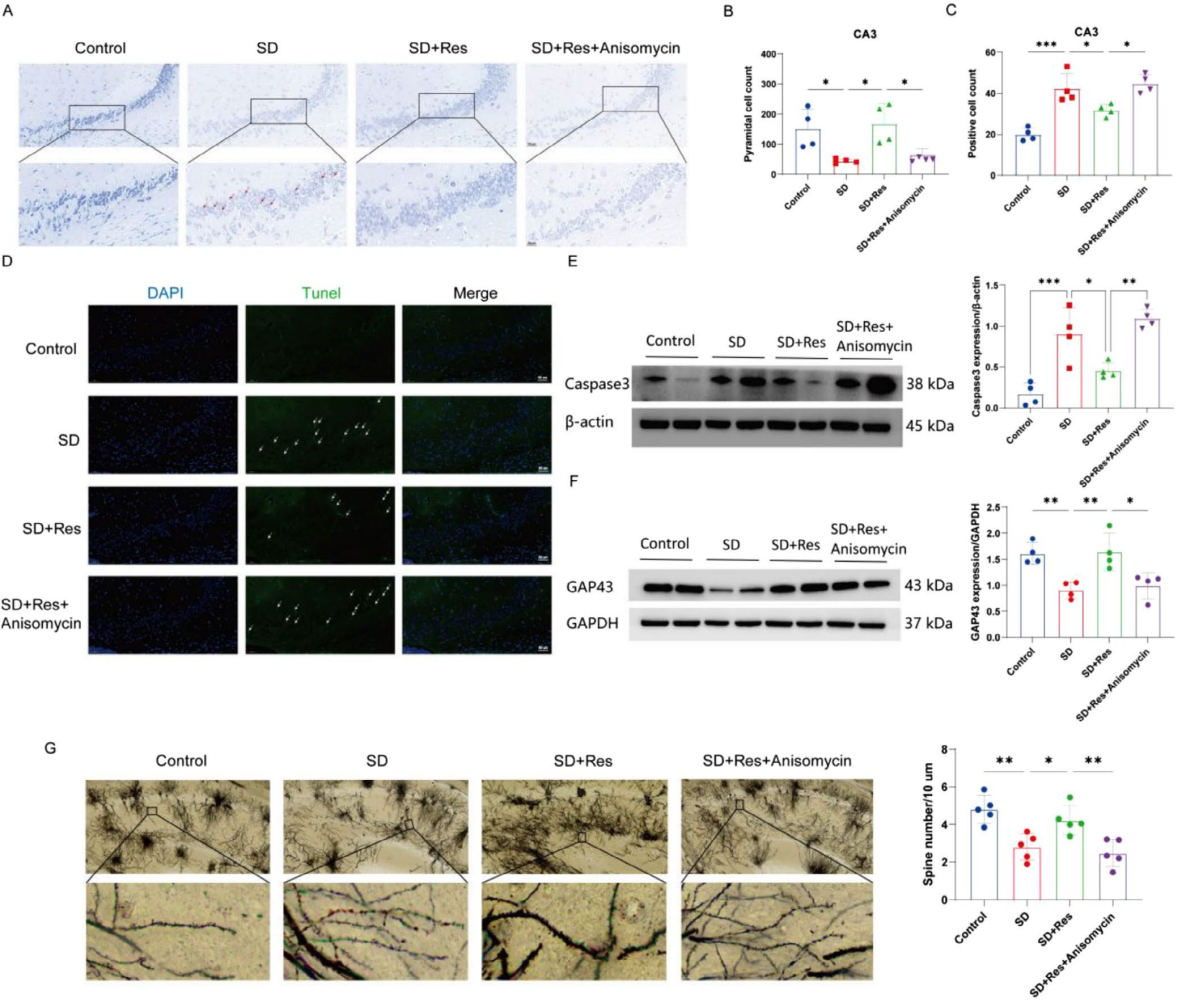
RES protects hippocampal neurons in sleep‐deprived mice by inhibiting p38 MAPK signaling pathway. (A, B) Anisomycin lost the effect of RES on reducing neuronal damage (*n* = 4). (C–E) RES reduced apoptosis in sleep‐deprived mice by inhibiting p38 MAPK signaling pathway (*n* = 4). (F) Synapse‐associated proteins GAP‐43 in hippocampus of mice, and RES pretreatment could prevent this effect. Anisomycin can disable this effect (*n* = 4). (G) Anisomycin reversed the improvement of RES on neuronal structure (*n* = 5; *p < 0.05, **p < 0.01, and ***p < 0.001).

The results of the Nissl staining showed that sleep deprivation led to a decrease in the number of pyramidal neurons in the CA3 region of the hippocampus. Anisomycin lost the effect of RES on reducing neuronal damage (Figure 7A,B). Tunnel fluorescence staining showed that the number of green fluorescent cells in the hippocampal CA3 region of the SD + RES + Anisomycin group increased, indicating that RES reduced apoptosis in sleep‐deprived mice by inhithe biting p38 MAPK signaling pathway (Figure 7C,D). Caspase‐3 was upregulated in the SD group compared to the Control group. The expression of Caspase3 was down‐regulated in the SD + RES group, anisomycin could reverse this effect of RES (Figure 7E). Sleep deprivation decreased the expression of synapse‐associated proteins GAP‐43 in hippocampus of mice, and RES pretreatment could prevent this effect. Anisomycin can disable this effect (Figure 7F). Anisomycin decreased the density of dendritic spines in pyramidal cells in CA3 region of the hippocampus, which reversed the improvement of neuronal structure induced by RES (Figure 7G).

## 4 Discussion

In our study, we found that following SD induction, cognitive function in mice was accompanied by apoptosis and changes in synaptic plasticity. RES can improve cognition by inhibiting the p38 MAPK signaling pathway. Therefore, this finding not only offers a novel perspective on preventing cognitive impairment in clinical settings but also supports the development of new pharmacological treatments.

Adequate sleep, a balanced diet, and regular exercise are the three health standards recognized by the international community. However, sleep deprivation is increasingly prevalent in today's fast‐paced society. The newly released “China Sleep Research Report 2022” shows that the average length of sleep nationwide has decreased from 8.5 h in 2012 to 7.06 h in 2021, with only 35% of the population achieving 8 h of sleep, and the adult insomnia rate has reached 38.2% (Yang et al. 1987). Sleep deprivation can cause numerous health issues, including cardiovascular diseases, diabetes, and obesity; particularly, cognitive dysfunction resulting from sleep deprivation has garnered significant societal concern due to its severity. In 2021, researchers from Harvard discovered that sleep deprivation can cause significant damage to the adolescent brain, leading to cognitive issues such as memory, attention, and mood disturbances (Brooks et al. 2022). Currently, clinical treatments for cognitive impairment resulting from sleep deprivation are scarce and limited; consequently, further research is essential to discover new therapeutic agents or tools. RES, a natural antioxidant, can serve as a raw material for health food products and the development of new drugs. It is commonly found in plants such as cassia, grapes, and tiger nuts. Existing studies have shown that RES exerts effects such as delaying aging, combating cardiovascular diseases, providing anti‐inflammatory and anti‐tumor benefits, and preventing Alzheimer's disease (Moussa et al. 2017; Chen et al. 2019; Li et al. 2021). Growing evidence suggests that phytonutrients or phytochemicals may have beneficial effects on cognitive impairment (Howes and Perry 2011; Mazzanti and Di Giacomo 2016; Wang et al. 2017), and RES is no exception.

This study focused on RES and sleep deprivation, utilizing a sleep deprivation chamber to simulate 72 h of acute sleep deprivation in mice, along with classical cognitive behavioral tests such as the Morris Water Maze and open field tests to assess the cognitive functions of the mice, including learning, memory, and mood. We observed that mice subjected to sleep deprivation exhibited reduced learning and memory capabilities, accompanied by signs of anxiety and depression‐like moods, consistent with findings from previous studies (Lin et al. 2024). Although numerous studies over the past few years have explored RES's effects on cognition, research into its impact on sleep deprivation‐induced cognitive impairment remains scarce. Therefore, in this study, mice in the experimental group received 100 mg/kg/day of RES via gavage for 14 days prior to sleep deprivation. It was observed that these mice did not exhibit cognitive impairment post‐sleep deprivation, suggesting that RES mitigates sleep deprivation‐induced cognitive deficits. Mice were administered resveratrol by gavage at a dose of 100 mg/kg per day for 14 consecutive days. Based on the body surface area conversion formula (Qi et al. 2025), the human equivalent dose is approximately 8.11 mg/kg. For a 60 kg adult, this corresponds to an estimated daily intake of about 487 mg of resveratrol. Given the very low content of resveratrol in food, it is difficult to achieve this dosage through diet alone; supplementation may therefore be a more feasible approach. It is noteworthy that although numerous animal studies have confirmed RES's diverse biological and pharmacological benefits, human clinical studies remain limited and controversial (Wilson et al. 1996; Crowell et al. 2004; Cucciolla et al. 2007; Guha et al. 2010; Carrizzo et al. 2013; Mankowski et al. 2020). The clinical efficacy of RES may vary due to differences in patient conditions, dosage, and treatment duration (Tomé‐Carneiro et al. 2013). Identifying the lowest effective and safe dose of RES remains a critical area for future research.

Current mechanisms by which sleep deprivation leads to cognitive dysfunction include enhanced inflammatory responses, altered synaptic plasticity, induced oxidative stress, modulated histone acetylation, abnormal neurotransmitter secretion, and increased cellular autophagy and apoptosis (Walker and Stickgold 2004; Wadhwa et al. 2017; Raven et al. 2018; Uddin et al. 2020). The neuroprotective mechanisms of RES remain undefined and may include inhibition of neuronal apoptosis, antioxidant and anti‐inflammatory effects, and modulation of synaptic plasticity (Moussa et al. 2017; Griñán‐Ferré et al. 2021). In this study, to elucidate the potential mechanisms by which RES ameliorates sleep deprivation‐induced cognitive impairment, mouse hippocampal tissues were analyzed using transcriptome sequencing. This analysis identified 29 genes altered by both sleep deprivation and RES treatment, associated with neuronal growth, development, and cell death functions.

Apoptosis is a tightly controlled multi‐protein process involving a series of proteins responsible for activation, expression, and regulation. Among these, proteins from the Caspase and Bcl‐2 families play crucial roles in apoptosis signaling. Caspases serve as initiators and executors of apoptosis in mammalian cells and are associated with CNS injuries. Bcl‐2 family proteins are categorized into two groups: anti‐apoptotic proteins, primarily including Bcl‐2 and Bcl‐w, and pro‐apoptotic proteins, primarily including Bax and Bak (Vandenabeele et al. 2023; Bashir et al. 2024). In this study, we analyzed the expression levels of hippocampal Caspase‐3, Bax, and Bcl‐2 proteins. We observed significant increases in these proteins following sleep deprivation, which were attenuated by RES pretreatment, thereby corroborating the sequencing results. This finding demonstrated that the inhibition of apoptosis is one mechanism by which RES alleviates sleep deprivation‐induced cognitive impairment.

Synaptic plasticity, closely related to learning and memory (Byrd and Brunjes 2001), includes structural and efficacy plasticity, both of which promote neural regeneration. GAP‐43, a presynaptic plasticity protein widely distributed in neurons, promotes neuronal growth, development, and axon regeneration (Shastry et al. 2007). SYN, a calcium‐binding acidic glycoprotein on presynaptic vesicle membranes, regulates synaptic transmission strength and neurotransmitter release through phosphorylation (Suzuki et al. 2020). PSD‐95, a postsynaptic plasticity protein, influences synaptic plasticity by modulating glutamate receptors. In this study, we assessed hippocampal GAP‐43, SYN, and PSD‐95 protein expressions. We noted that GAP‐43 expression was down‐regulated in sleep‐deprived mice, a change preventable by RES pretreatment. However, SYN and PSD‐95 expressions did not show statistically significant changes in the hippocampal region post‐sleep deprivation. This suggests that RES may attenuate cognitive impairment in sleep‐deprived mice by modulating synaptic plasticity, primarily targeting structural aspects such as hippocampal neuronal growth. Additionally, the morphological effects of RES pretreatment on the number and morphology of synaptic dendritic spines in hippocampal neurons were further verified through Golgi staining.

Functional enrichment analysis of the 29 significantly differentially expressed genes identified in transcriptome sequencing revealed associations with apoptosis and synaptic plasticity, and also highlighted the MAPK signaling pathway, a classical signaling route. MAPK consists of evolutionarily conserved serine/threonine protein kinases, activated by various extracellular stimulatory signals. These kinases mediate signaling from the cell membrane to the nucleus and regulate processes such as proliferation, differentiation, and cell death, influencing physiological and pathological phenomena including inflammation, apoptosis, tumor invasion, and metastasis. P38 mitogen‐activated protein kinase (P38 MAPK) is a key member of the MAPK family (Wang et al. 2024). Relevant studies have shown that P38 is closely associated with apoptosis and promotes it (Sui et al. 2014). Furthermore, P38 has been demonstrated to be activated by pro‐inflammatory factors, subsequently affecting neuronal synaptic function (Tancredi et al. 1992; Kelly et al. 2003). Consequently, we hypothesize that sleep deprivation may promote apoptosis and impair synaptic function by activating the P38 MAPK signaling pathway. RES could inhibit this apoptosis and modulate synaptic plasticity by inhibiting P38 phosphorylation, suggesting that MAPK may be a target of RES's action. This study confirmed that sleep deprivation activates the P38 signaling pathway, which can be inhibited by RES, aligning with previous findings. To further substantiate that MAPK mediates sleep deprivation‐induced hippocampal neuronal apoptosis and reduced synaptic plasticity, this study employed anisomycin in subsequent experiments for verification.

This study has some limitations, including the lack of further validation of hippocampal functional plasticity using electrophysiological methods. The clinical significance of studying acute sleep deprivation (such as short‐term deprivation within 72 h) differs markedly from that of chronic sleep deprivation (characterized by insufficient sleep over several weeks or months). The former focuses more on the immediate effects of sudden sleep loss, modeling of pathological mechanisms, and emergency intervention strategies, whereas the latter emphasizes the long‐term cumulative damage associated with chronic insufficiency. Therefore, the acute sleep deprivation model cannot replicate the conditions of chronic sleep deprivation. In the future, we will further investigate the neuroprotective effects of resveratrol in the context of chronic sleep deprivation. There remains a significant gap between mechanistic exploration and clinical translation in this study. This gap not only reflects the current limitations of the research but also highlights key directions for future breakthroughs. Innovative clinical study designs are needed to shift from reliance on animal models to the collection of real‐world evidence for clinical application.

## 5 Conclusions

In conclusion, this study demonstrated for the first time the beneficial effects of RES in a sleep deprivation model, providing a novel and effective therapeutic approach for preventing and improving sleep deprivation‐induced cognitive dysfunction, which holds significant clinical potential. Furthermore, this study employed sequencing technology and contemporary bioinformatics analysis to investigate RES's mechanism, discovering that it ameliorates sleep deprivation‐induced cognitive impairment by inhibiting apoptosis and enhancing synaptic plasticity. It also identified the p38 MAPK signaling pathway as a target of RES, offering theoretical support for its future clinical applications. In summary, this study offered theoretical support that RES has the potential to be a dietary supplement with cognition‐improving effects.

## Author Contributions

**Yijie Tang:** formal analysis (equal), funding acquisition (equal), investigation (equal), writing – original draft (equal), writing – review and editing (equal). **Xiyuan Xie:** investigation (equal), methodology (equal), writing – original draft (equal), writing – review and editing (equal). **Haixing Wu:** writing – original draft (equal), writing – review and editing (equal). **Xinlei Huang:** data curation (equal), formal analysis (equal), investigation (equal), validation (equal), visualization (equal), writing – original draft (equal). **Jiapeng Qiu:** formal analysis (equal), methodology (equal), supervision (equal), validation (equal), visualization (equal). **Yupeng Han:** conceptualization (equal), data curation (equal), resources (equal), software (equal), validation (equal), visualization (equal). **Peng Ke:** investigation (equal), methodology (equal), software (equal), supervision (equal), validation (equal), visualization (equal). **Kai Zeng:** funding acquisition (equal), project administration (equal). **Xiaodan Wu:** data curation (equal), funding acquisition (equal), project administration (equal).

## Ethics Statement

All experimental procedures and housing conditions for animals were in compliance with the Guide of the National Institutes of Health (NIH) for the Care and Use of Laboratory Animals and approved by the Laboratory Animal Ethics Committee of Fujian Medical University (IACUC FJMU 2022‐0889).

## Consent

The authors have nothing to report.

## Conflicts of Interest

The authors declare no conflicts of interest.

## Supporting information


**Table S1:** Twenty‐nine significant DEGs were reversed by RES treatment.

## Data Availability

The original data can be acquired by connecting to the corresponding author.
